# Versatile Biodegradable Poly(acrylic acid)-Based Hydrogels Infiltrated in Porous Titanium Implants to Improve the Biofunctional Performance

**DOI:** 10.1021/acs.biomac.3c00532

**Published:** 2023-09-07

**Authors:** Guillermo Martínez, Belén Begines, Eloisa Pajuelo, Juan Vázquez, Luisa Marleny Rodriguez-Albelo, Davide Cofini, Yadir Torres, Ana Alcudia

**Affiliations:** † Departamento de Química Orgánica y Farmacéutica, Facultad de Farmacia, Universidad de Sevilla, Seville 41012, Spain; ‡ Departamento de Microbiología y Parasitología, Facultad de Farmacia, Universidad de Sevilla, Seville 41012, Spain; § Departamento de Química Orgánica, Facultad de Química, Universidad de Sevilla, Seville 41004, Spain; ∥ Departamento de Ingeniería y Ciencia de los Materiales y del Transporte, Escuela Politécnica Superior, Universidad de Sevilla, Seville 41011, Spain

## Abstract

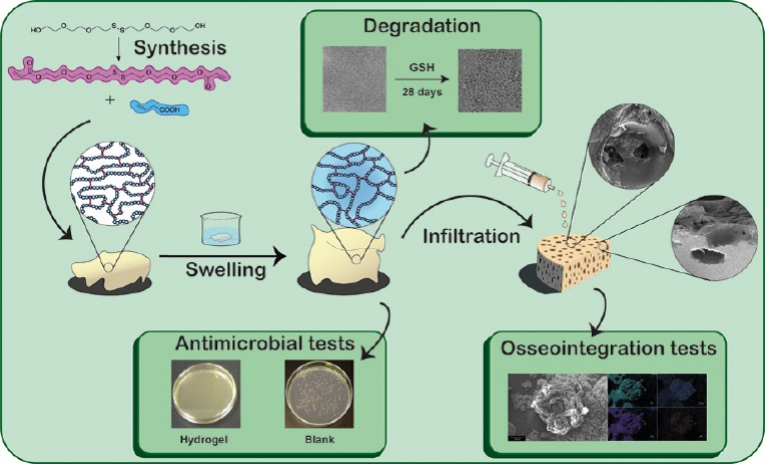

This research work proposes a synergistic approach to improve implants’ performance through the use of porous Ti substrates to reduce the mismatch between Young’s modulus of Ti (around 110 GPa) and the cortical bone (20–25 GPa), and the application of a biodegradable, acrylic acid-based polymeric coating to reduce bacterial adhesion and proliferation, and to enhance osseointegration. First, porous commercially pure Ti substrates with different porosities and pore size distributions were fabricated by using space-holder techniques to obtain substrates with improved tribomechanical behavior. On the other hand, a new diacrylate cross-linker containing a reduction-sensitive disulfide bond was synthesized to prepare biodegradable poly(acrylic acid)-based hydrogels with 1, 2, and 4% cross-linker. Finally, after the required characterization, both strategies were implemented, and the combination of 4% cross-linked poly(acrylic acid)-based hydrogel infiltrated in 30 vol % porosity, 100–200 μm average pore size, was revealed as an outstanding choice for enhancing implant performance.

## 1 Introduction

Since life expectancy has increased greatly over the past decades, the lifespan of organs and systems in the human body remains one of the major health problems. In the medical industry, a large number of implants, prostheses, and medical devices have been developed to recover the functionality of human organs to improve the quality of life of patients affected by a wide range of diseases or physical ailments.^1^ These devices have been designed to include exacting standards to fit in regions with high chemical or electrical activity, such as neuroprosthetics and monitoring devices^2,3^ or to meet requirements and support high mechanical stress, such as bone replacement in joints or maxillofacial prosthesis. In this sense, biocompatible metals and their alloys have previously been applied in the fabrication of implants as bone substitutes. Among these metals, Ti and its Ti6Al4 V alloy are the most applied in implantology^4−6^ due to their excellent biocompatibility, exceptional corrosion resistance, and high specific mechanical properties. However, unfortunately, they present recognized problems that can compromise the clinical performance of the prosthesis over time: (1) the mismatch between Young’s modulus values of Ti and Ti6Al4 V alloy (around 110 GPa) and cortical bone (20–30 GPa), also known as the stress-shielding phenomenon, has been identified as a main reason for implant loosening and bone resorption;^7,8^ (2) when the Ti6Al4 V implant is placed into the body, the alloying elements aluminum and vanadium, which have toxic effects on the brain (Alzheimer’s disease) and body, respectively, could be released as metallic ions;^9^ (3) their biocompatible surface favors adhesion and proliferation of bacteria, leading to microbial-related infections; and (4) poor osseointegration inherent to metallic implants inhibits the formation of new bone, forming a thin fibrous layer, with subsequent encapsulation and loosening of the implant (medium and long term). This research work presents a synergistic approach to overcome these issues that address two strategies.

On the one hand, the implementation of porous Ti implants could be a possible solution^10,11^ to reduce the stress-shielding phenomenon. The use of space-holder materials (such as carbamide,^12,13^ NaCl,^14,15^ K_2_CO_3_,^16^ and poly(methyl methacrylate)^17^) has recently been developed and spread, with numerous advantages (adjustable porosity amount, great uniformity, controlled pore shape, and more uniform pore size distribution^18,19^) compared to traditional challenges.^18,20^ However, Ti-based implants still need to improve their osseointegration or antibacterial performance to implement bioactivity.^20,21^ On the other hand, these limitations will be addressed by applying polymeric-based coatings with demonstrated antibacterial capacity and osseointegration induction.

Currently, hydrogels are gazed on as emerging innovative materials with optimal characteristics for tissue mimicking,^22−24^ tissue engineering,^25−27^ and bone regeneration.^28−30^ The chemical composition of hydrogels, by modifying both the polymeric chain or the cross-linking agent, can be tuned to design a specific material behavior (strength, elasticity, or bioactivity).^31−34^ A tunable polymer structure with fragments containing disulfide bonds could be an optimal way to turn a hydrogel into biodegradable, as disulfide bonds are reduced by biomolecules such as glutathione, a ubiquitous molecule within the human body.^35,36^ In addition to that, the ability of hydrogels to mimic the environment of the tissue material is of special interest to promote cell adhesion and proliferation,^37,38^ as they can even be functionalized with peptides and other biomolecules to improve specific cell growth.^39−41^ Furthermore, some of these materials possess intrinsic antibacterial activity, making them an excellent choice for their application in wound healing or implant devices.^42−44^ In this sense, poly(acrylic acid) is a biodegradable, nontoxic polymer, which has experienced a high increase on its use for a wide variety of biomedical applications.^45,46^ On its own or in combination with other polymers, it has been used in nanocomposites, drug delivery systems, as mucoadhesive hydrogels for wound healing, etc.^45,47,48^ Furthermore, poly(acrylic acid) possesses high versatility, thanks to its high hydrophilicity and superabsorbent capacities, making this polymer a great option when designing bioactive and biocompatible hydrogels for biomedical purposes.^49−51^ In recent years, hydrogels have been widely studied and developed for these proposals, since little or even no side effects have been described for these materials compared to traditional antibacterial therapeutic agents, and do not trigger antibacterial resistance.^52−54^ However, the mechanisms of this intrinsic antimicrobial behavior are different in nature, which, along with the wide variety of hydrogels and their insolubility, make it difficult to study their properties.^55−57^ In addition, the liquid absorption determines the capacity of hydrogels to form a releasing matrix or synergic composite,^58^ where therapeutic agents are embedded inside the hydrogel and released over a period of time, so that hydrogel can be adapted with great versatility and adaptability to several circumstances and situations.^59,60^

Therefore, in summary, to overcome the previously mentioned limitations of metallic implants, the use of porous commercially pure (c.p.) Ti is proposed together with the application of a novel antibacterial and degradable osseointegrative hydrogel coating based on poly(acrylic acid). The space-holder technique is employed to obtain two types of porous c.p. Ti substrates^19^ with different tribomechanical performance. The porosity, shape, and pore size distributions of the samples are described. A novel synthesis diacrylate cross-linker containing a degradable disulfide bond^61,62^ is obtained, purified, and employed to prepare poly(acrylic acid)-based hydrogels with desirable degradation properties in the presence of glutathione,^36^ allowing the polymeric chain to potentially disappear as the osseointegration occurs. Swelling capacity, degradation, thermogravimetric analysis, wettability, antimicrobial properties against Gram-positive (*Staphylococcus aureus*) and Gram-negative (*Pseudomonas aeruginosa*) type strains of bacteria, and osseointegration in vitro studies are described^57,63^ for these coating materials.^64,65^

## 2 Materials and Methods

**Figure 1:**
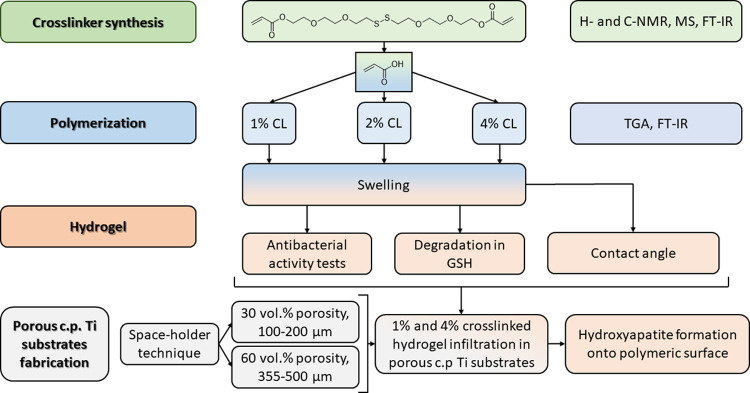
Schematic of the experimental design carried out to develop this research work. Initially, a novel disulfide-based monomer was synthesized to be used as a cross-linker in the preparation of poly(acrylic acid)-based hydrogels containing 1, 2, and 4% of the cross-linker. Polymers were characterized including their swelling capacity. Then, hydrogels were synthesized adding the maximum amount of water they can absorb since they were going to be infiltrated in their swelled form. Antibacterial, degradation, and contact-angle tests were conducted to characterize the materials considering their final application. On the other hand, porous c.p. Ti substrates were fabricated following the space-holder technique to achieve highly differentiated pore contents and pore size distributions according to the results previously published by the authors.^66−69^ Then, hydrogels with 1 and 4% cross-linker were infiltrated on top of the c.p. Ti substrates using a heat-shrink tube and the hydroxyapatite formation capacity of infiltrated hydrogels was investigated. Finally, the best tandem porous substrate-hydrogel was selected considering a balance among lower porosity of the substrate, better infiltration, and better bifunctional behavior.

Figure 1 shows the schematic workflow of the experimental protocols for this investigation. Cross-linker synthesis, poly(acrylic acid) polymerization (with comparable cross-linker content), and hydrogel formation, including characterization of all species, were performed to search for the best chemical entity in terms of swelling, degradation, and antibacterial properties against different strains. The selected hydrogels were infiltrated in porous c.p. Ti substrates with different pore sizes, and the content was fabricated using the space-holder technique, suitable for its biomechanical and biofunctional balance, as demonstrated in previous studies.^8,66^ In addition, infiltrated porous substrates were further explored for the evaluation of osseointegration as a critical therapeutic implant requirement.

### 2.1 Materials and General Characterization Methods

Triethylene glycol monochlorohydrin (96%), acryloyl chloride (≥97%), acrylic acid, and triethylamine (≥99.5%) were purchased from Sigma-Aldrich Pvt. Ltd. (Madrid, Spain). Reduced l-glutathione (≥98%) was purchased from Thermo Fisher Scientific Inc. (Lancashire, United Kingdom). Dichloromethane (DCM), methanol (MeOH), hexane (Hex), and ethyl acetate (AcOEt) solvents were purchased from Scharlab S.L.Commercial (Barcelona, Spain). All chemicals were used without further purification. Pure Ti powder with a mean particle size of *d*_[50]_ = 23.3 μm^58^ was provided by SEJONG Materials Co. Ltd. (Seoul, Korea). Ammonium bicarbonate with a purity of 99% was supplied by Cymit Química S.L. (Barcelona, Spain). For antimicrobial experiments, tryptone soy agar (TSA) was purchased from Merck, and tryptone soy broth (TSB) was purchased from Liofilchem S.r.l. (Barcelona, Spain). Type strains *P. aeruginosa* (CECT 108) and *S. aureus* (CECT 5190) were purchased from the Spanish Type Culture Collection (CECT). NMR spectra were recorded at 300 K on a Bruker Advance AV-500 instrument or a Bruker AMX-500. The mass spectrum was obtained in a Thermo Scientific Orbitrap Elite. Thermogravimetric analysis (TGA) was performed under a nitrogen atmosphere (flow rate 100 mL/min) with a TA Instruments SDT Q600 at a heating rate of 10 °C min^–1^. IR spectra were recorded on a Jasco FT/IR 4200 spectrometer equipped with an ATR. Degradation tests were carried out in a Heidolph Unimax 1010 system with controlled temperature. Osseointegration in simulated body fluid (SBF) tests was performed in a Digitheat-TFT desiccation oven. SEM images and elemental composition analysis were obtained in an FEI Teneo or Zeiss Auriga microscope, respectively.

### 2.2 Synthesis of Cross-Linker

**Figure 2:**
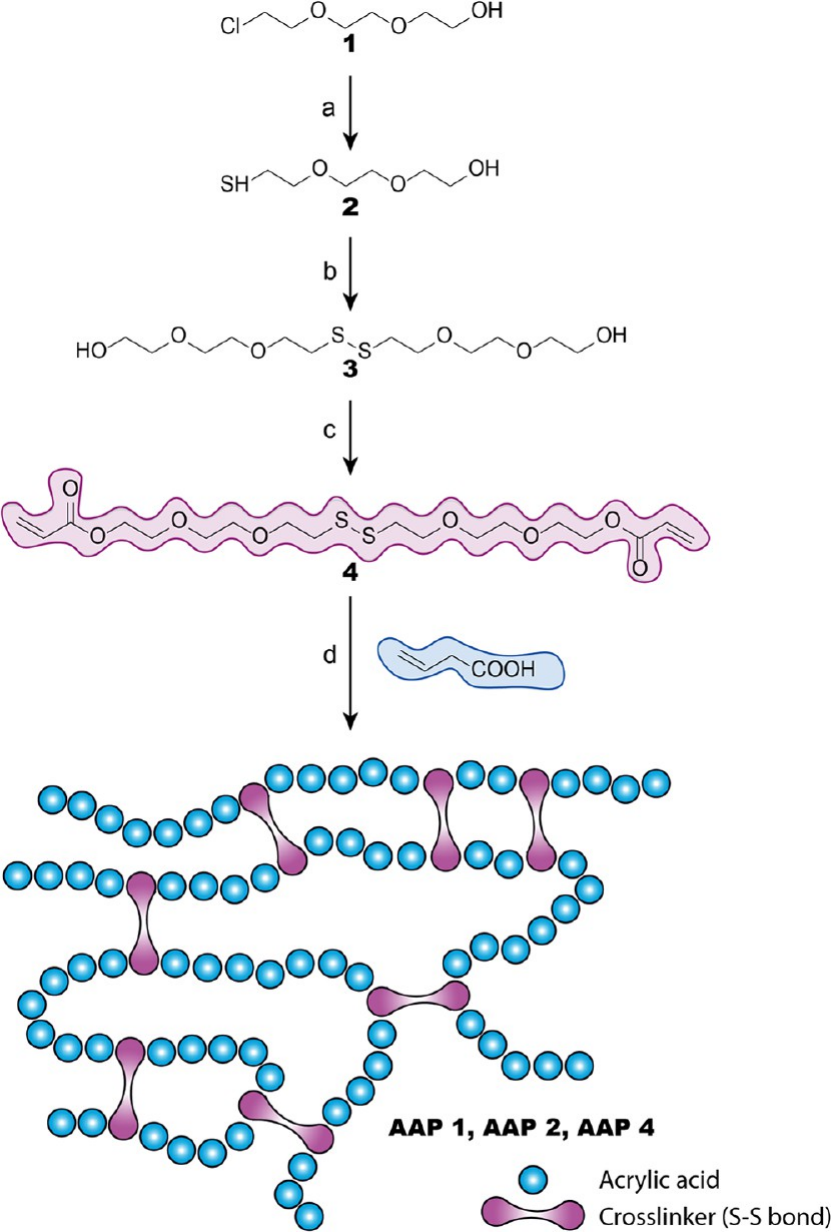
Schematic of the synthetic route of cross-linker and polymers. (a) NaHS, HCl, EtOH, 60 °C; (b) I_2_, K_2_CO_3_, MeOH; (c) acryloyl chloride, triethylamine, DCM, 0 °C; and (d) acrylic acid, azobis(isobutyronitrile) (AIBN), 65 °C.

The preparation of the cross-linker was carried out following the synthetic route depicted in Figure 2.

#### 2.2.1 2-(2-(2-Mercaptoethoxy)ethoxy)ethan-1-ol^70^ (**2**)

This compound was prepared by dissolving 10 g (60 mmol) of triethylene glycol monochlorohydrin (1) and 22.2 g (300 mmol) of sodium hydrogen sulfide monohydrate in 200 mL of ethanol and heated to 60 °C. A mixture of 15 mL of concentrated HCl and 100 mL of ethanol was then added dropwise over a period of 6 h. The product obtained was filtered and concentrated at 40 °C. The residue was dissolved in cold ethanol and filtered again to obtain a yellow oil after evaporation of the solvent. To eliminate by-products, the oil was completely dissolved in DCM and purified using DCM–MeOH (50:1) as the eluent. This implementation replaced the distillation process described by the authors. The final product **2** was obtained as a yellowish oil with a quantitative yield (99% yield).

#### 2.2.2 3,6,13,16-Tetraoxa-9,10-dithiaoctadecane-1,18-diol^70^ (**3**)

3.6 g (21,6 mmol) of **2** was dissolved in 100 mL of methanol and mixed with 50 mL of a potassium carbonate solution (0.23 M). 2.77 g (10.8 mmol) of I_2_ in 100 mL of methanol was poured dropwise at room temperature. Then, small portions of sodium sulfite were added until discoloration. After the solution was evaporated to dryness, the residue was suspended in DCM and the potassium iodide was removed by filtration after cooling. The resulting solution was evaporated once again. To eliminate the inorganic salt, the product was filtered and dried under vacuum, yielding a nearly colorless oil (73% yield). As an improvement to the previous methodology, no purification process was carried out at this point.

#### 2.2.3 3,6,13,16-Tetraoxa-9,10-dithiaoctadecane-1,18-diyl Diacrylate (**4**)

Compound **3** (250 mg, 0.75 mmol) was added to a round-bottom flask, and a vacuum-argon cycle was applied in triplicate. Then, DCM (3 mL) and triethylamine (227.7 mg, 465 μL, 2.25 mmol) were added and the mixture was stirred for 30 min. Subsequently, acryloyl chloride (203.7 mg, 183 μL, 2.25 mmol) was added, and the solution was cooled to 0 °C. Finally, the reaction mixture was left to stir overnight. The reaction mixture was then treated with 0.1 M K_2_CO_3_ solution, and the organic phase was dried with MgSO_4_, filtered, and concentrated under reduced vacuum. The crude product was purified by column chromatography using AcOEt–Hex (2:1). The final product **4** was obtained as a light yellowish oil (52% yield).

##### 2.2.3.1 ^1^H NMR (CDCl_3_, 500 MHz)

δ (ppm) 2.88 (t, 4H, H-8, H-8′, *J* = 6.7 Hz), 3.63–3.66 (m, 8H, H-5, H-5′, H-6, H-6′), 3.72–3.75 (m, 8H, H-4, H-4′, H-7, H-7′), 4.30–4.32 (m, 4H, H-3, H-3′), 5.82–5.84 (dd, 2H-1a, *J*_H-1a,H-1b_ = 1.5 Hz, *J*_H-1a,H-2_ = 10.5 Hz), 6.12–6.18 (dd, 2H-2, *J*_H-1a,H-2_ = 10.5 Hz, *J*_H-1b,H-2_ = 17.3 Hz), 6.40–6.44 (dd, 2H-1b, *J*_H-1b,H-2_ = 17.3, *J*_H-1a,H-1b_ = 1.5 Hz). ^13^C NMR (CDCl_3_, 500 MHz): δ (ppm) 38.5 (C-8), 63.6 (C-3), 69.2 (C-7), 69.7 (C-6), 70.41 (C-5), 70.58 (C-4), 128.3 (C2=C1), 130.95 (C1=C2), 166.13 (C=O). IR: ν (cm^–1^) 2868 (C–H); 1720 (C=O); 1188 (C–O–C); 984 (C = CH). HRFABMS: Calculated molecular weight for C_18_H_30_O_8_NaS_2_: (M + Na)^+^ 461.1301; experimental molecular weight: 461.1274

### 2.3 Synthesis and FTIR Characterization of Poly(acrylic acid)-Based Polymers (Dry Polymeric Materials)

A general polymerization procedure for synthesizing each polymer is described in detail. An amount of acrylate was poured into a vial, followed by the addition of **4** (1, 2, or 4% w/w) and azobis(isobutyronitrile) (AIBN) (1% w/w). The mixture was then dissolved. Vacuum-argon cycles were applied in triplicate to produce an inert atmosphere. The mixture was heated to 60 °C for 15 min to obtain colorless polymers: 1% cross-linked poly(acrylic acid) (**AAP1**), 2% cross-linked poly(acrylic acid) (**AAP2**), and 4% cross-linked poly(acrylic acid) (**AAP4**).

#### 2.3.1 Polymers **AAP**

Polymer AAP1: IR: ν (cm^–1^) 3532 (O–H)_intermolecular_; 3203 (O–H); 2933 (C–H); 1702 (C=O); 1157 (C–O). Polymer **AAP2**: IR: ν (cm^–1^) 3463 (O–H)_intermolecular_; 3182 (O–H); 2934 (C–H); 1703 (C=O); 1159 (C–O). Polymer **AAP4**: IR: ν (cm^–1^) 3473 (O–H)_intermolecular_; 3178 (O–H); 2932 (C–H); 1702 (C=O); 1158 (C–O).

### 2.4 Swelling Tests of Poly(acrylic acid)-Based Polymers

Swelling tests were performed to define the maximum capacity of water absorption of polymers prior to the preparation of the corresponding hydrogels. In this sense, five samples of each polymer (AAP1, AAP2, and AAP4) were prepared, weighed, and submerged in distilled water. The weight was measured after 3, 6, 12, 24, and 48 h of immersion. The swelling capacity was calculated using eq 1
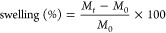
1where *M_t_* stands for polymer mass after *t* hours of immersion (swelled) and *M*_0_ stands for dried polymer mass.

### 2.5 Synthesis of Poly(acrylic acid)-Based Hydrogels

Three hydrogels (AAH1, AAH2, and AAH4) were prepared in a similar way as previously described for polymers AAP1–AAP4 but adding an amount of distilled water according to the previously investigated swelling equilibrium, being 73.5% w/w of total polymer weight for 1% cross-linked poly(acrylic acid) hydrogel (AAH1), 68.8% w/w of total polymer weight for 2% cross-linked poly(acrylic acid) hydrogel (AAH2), and 59.2% of total polymer weight for 4% cross-linked poly(acrylic acid) hydrogel (AAH4).

### 2.6 Degradation of Hydrogels in Reductive Environment

To assess the degradation rate of the as-prepared polymeric materials, the degradability was estimated in a reductive environment in the presence of glutathione. Hydrogels were introduced into flasks and submerged in a solution of 10 mM GSH, the reduced form of glutathione,^36,71^ in distilled water under an inert atmosphere to avoid oxidation by O_2_. Every 2 days, the GSH solution was carefully removed and replaced with a fresh solution. The degradation was investigated by SEM after 28 days of immersion. In addition, a study of the porosity evolution was conducted in the three hydrogels using the software ImageJ.

### 2.7 Wettability Studies of Hydrogels

To evaluate the wettability of the materials, static contact-angle measurements (Phoenix 300 Touch Automatic Contact Angle Analyzer, SEO) were conducted by depositing a macroscopic droplet of distilled water. Average values were obtained from five replicates applying the Young’s equation with the software Surfaceware 7.^72−74^

### 2.8 Fabrication of Porous Ti Samples

**Figure 3:**
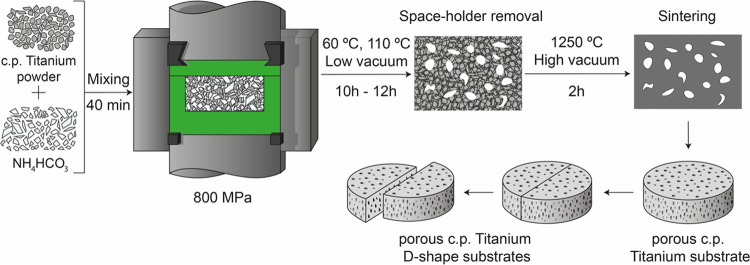
Ti substrate fabrication process scheme.

Porous substrates used in this study were obtained by a space-holder technique. A grade IV commercial pure titanium powder (c.p. Ti) with a mean powder size of *d*_[50]_ = 23.3 mm was mixed with different percentages (30 and 60 vol %) and ranges of particle sizes (100–200 and 355–500 μm) of ammonium bicarbonate (NH_4_HCO_3_). In this work, porous Ti substrates were selected following the below criteria: (1) those of 30 vol % (100–200 mm) were chosen to avoid loss of mechanical resistance, although being detrimental to the infiltration of the hydrogel, and (2) 60 vol % (355–500 mm) was selected to favor infiltration, despite being aware of the potential loss of mechanical performance. However, as discussed further in the next section, biopolymer infiltration plays an important role in improving the service efficiency. The mixture of titanium powder and spacer particles was homogenized in a Turbula T2C shaker-mixer for 40 min and pressed at 800 MPa with the same equipment. The NH_4_HCO_3_ was then removed in an oven at 10^–2^ mbar: first, at 60 °C for 12 h and second, at 110 °C for 12 more hours. Finally, the porous green discs were also sintered in a molybdenum chamber furnace at 1250 °C for 2 h, under high-vacuum conditions (10^–5^ mbar). Discs of ∼12 mm diameter and ∼2 mm height were obtained. Figure 3 displays a schematic of the applied fabrication process. To perform the different studies, the surfaces of porous substrates were prepared following a standard metallography procedure on one side of the discs (grinding and mechanical–chemical polishing). The porosity of all obtained substrates was studied by Archimedes’ method and image analysis (IA). The equivalent pore diameter, pore shape factor, and total and interconnected porosity (*D*_eq_, *F*_f_, *P*_T_, and *P*_i_, respectively)^75,76^ were evaluated by these methods. The image analysis has been carried out with at least five pictures of 5× for each type of substrate. Finally, the mechanical behavior of the porous substrates (dynamic Young’s modulus, *E*_d_, yield strength, and σ_*y*_) was estimated from the experimental porosity results (at least three measurements for each processing condition) and using fit equations reported in the literature.^77^

### 2.9 Evaluation of the Antibiofouling Capacity of Poly(acrylic acid)-Based Hydrogels

**Figure 4:**
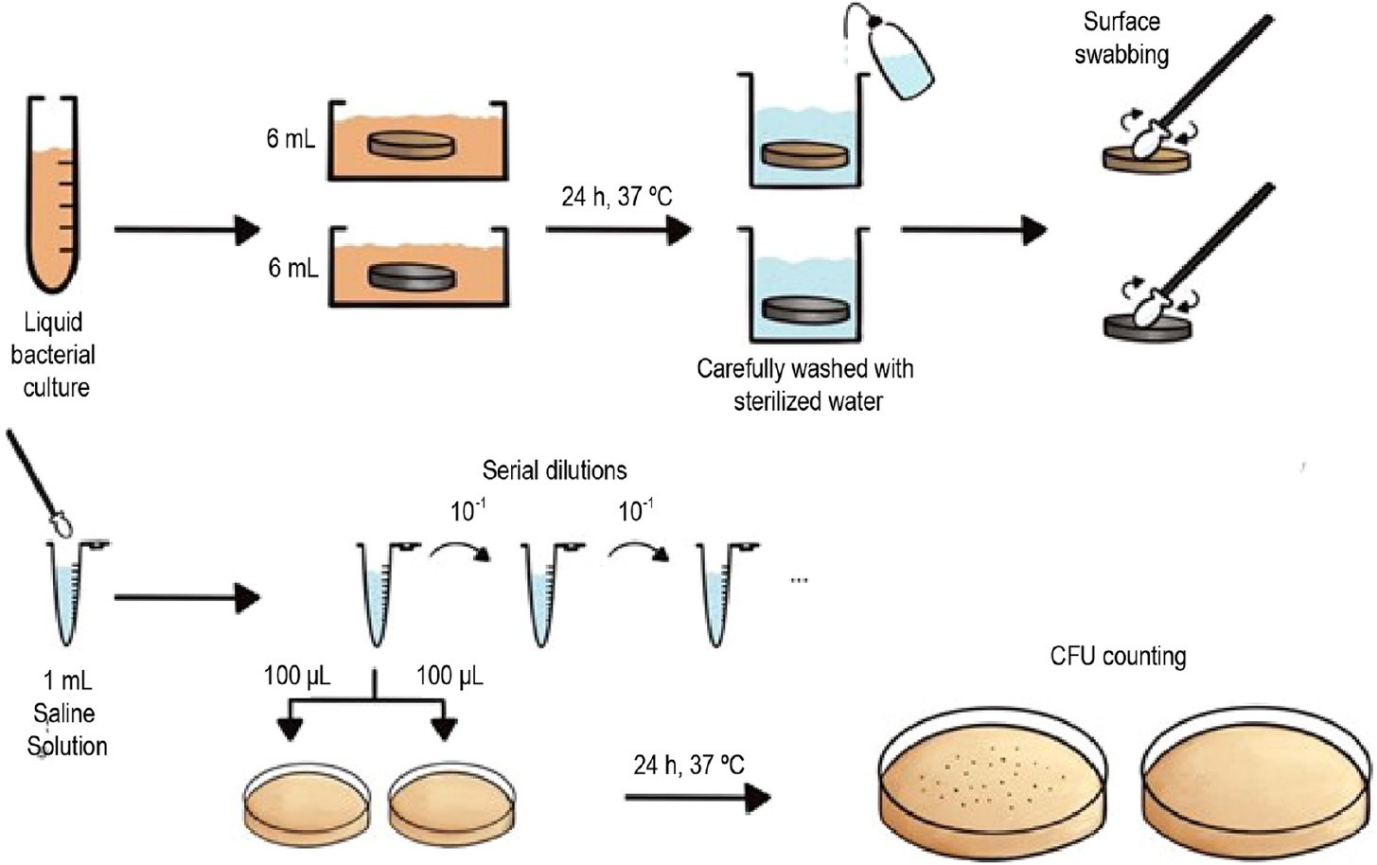
Diagram illustrating the designed bacterial experiment of hydrogels and c.p. Ti substrates to determine their antibacterial properties.

Two representative species of bacteria were used to evaluate the antibacterial behavior of the hydrogels: *S. aureus* (ATCC 25923) as a Gram-positive bacterium and *P. aeruginosa* (ATCC 27853) as a Gram-negative bacterium. In order to evaluate the potential activity as surface growth inhibitors of pathogen species of bacteria shown by hydrogels, the following methodology (Figure 4) was set and tuned up.

A bacterial suspension was first prepared from a single individual bacterial colony and inoculated in TSB medium. Initial bacterial concentrations were 8.62 × 10^5^ CFU/mL for *P. aeruginosa* and 5.67 × 10^5^ CFU/mL for *S. aureus*, where CFU is defined as colony-forming units. For the comparison of the potential improvement in the antibacterial capacity induced by hydrogels, two different types of samples were analyzed in this experiment: hydrogel cylinders were synthesized with dimensions of approximately 15 mm diameter and 5 mm height, while porous c.p. Ti cylinders were fabricated with a diameter of 12 mm and a height of 5 mm. However, the same procedure was applied for both types of samples, independent of their nature. A volume of 6 mL (enough to completely submerge the samples) of these bacterial suspensions was poured in each well of a six-well plate, where the samples were submerged. The bacteria were incubated for 24 h at 37 °C, and then the samples were removed from the TSB and carefully washed with sterile distilled water to remove the nonattached bacteria on their surface. As a control, samples were submerged in a sterile TSB medium. All experiments were performed in triplicate. Sterile swabs were used to wipe the entire surface of the samples to collect the attached bacteria. The tips of the swabs were removed with sterile scissors, introduced into Eppendorf tubes containing 1 mL of saline solution (0.9% NaCl), and washed for 5 min with gentle shaking. Serial dilutions (from 10^–1^ to 10^–5^) were performed in sterile saline solution and 100 μL of each dilution was striked out on TSA plates. After incubation of the plates for 24 h at 37 °C, bacterial colonies were counted for the determination of UFC mL^–1^. Finally, the bacterial density was expressed as CFU cm^–2^ for the surface of the cylinders. Hydrogels were tested against a c.p. Ti surface, referred to as blank, to determine whether hydrogels improve this property or not.

### 2.10 Visualization of Bacterial Attachment to Surfaces

The surfaces of hydrogel cylinders and porous c.p. and Ti cylinders (both, with 30% porosity and 100–200 μm pore size distribution, and with 60% porosity and 355–500 μm pore size distribution) were observed by low-vacuum scanning electron microscopy in order to visualize bacterial attachment. For this purpose, discs of the three materials (in duplicate) were submerged in volumes of 6 mL of the bacterial cultures (all of them at an optical density of 1.0 at 600 nm) for 24 h at 37 °C as described above. After incubation, the discs were washed three times with sterile distilled water to remove unattached bacteria. Samples were deposited on the circular sample devices of the microscope, frozen at −20 °C, and observed under low vacuum using a Phenom Pro microscope at the Microscopy Service of the CITIUS (Center for Research, Technology and Innovation, University of Seville, Spain).

### 2.11 Hydrogel Infiltration

Cross-linked poly(acrylic acid) hydrogels were prepared in situ on c.p. Ti samples fabricated as previously described. A homogeneous solution of the hydrogel components was prepared in distilled water under inert atmosphere. Then, 300 μL was poured into a porous c.p. Ti substrate sealed with a heat-induced shrinking material, and heat (60 °C) was applied for 30 min to activate the AIBN initiator and thus initiate the polymerization. Depending on the experiments to be conducted, the hydrogels were infiltrated in both complete substrates and D-shaped substrates. These last samples allowed the investigation of the penetration capacity of hydrogels into the inner pores of the substrates.

### 2.12 Hydroxyapatite Formation on Coated Substrates

An in vitro evaluation for the hydroxyapatite-forming ability of these materials in the presence of SBF was performed following ISO 23317:2014. Hydrogels AAH1 and AAH4 were infiltrated in c.p. Ti substrates and soaked in SBF for 28 days at 36.5 ± 2.0 °C. The SBF solution was renewed after 7, 14, and 21 days. On day 28, samples were taken out, gently but carefully washed with distilled water and observed by SEM in a Zeiss Auriga in order to determine the existence of hydroxyapatite and/or its precursor species nucleated on the polymeric surface. An elemental composition analysis was carried out to semiquantitatively determine the amount of Ca and P species. Some samples needed to be covered with a tiny Au film by sputtering with an Edwards Scancoat machine.

## 3 Results and Discussion

As previously mentioned, the main aim of this research work was to develop enhanced porous Ti-based implants for partial or complete bone substitutions that potentially improve the performance and limitations of the already-commercialized ones. This objective was approached by addressing two different strategies at the same time. On the one hand, the use of Ti with controlled porosity was proposed as the optimal option to reduce implant stiffness, to make it closer to the natural bone one, and therefore to reduce the stress-shielding phenomenon, one of the main causes of mechanical failure of implants.^5,78^ However, the introduction of pores into the substrates must be adequate to obtain not only the required biomechanical balance but also good biofunctional behavior, enhancing the vascularization of the implant and bone in-growth and improving the infiltration and adhesion of coatings. On the other hand, the second strategy was focused on the improvement of other characteristics related to implant loosening such as bacterial proliferation or poor osseointegration.^8,63^ By coating the implant, including the pores, the mechanical, corrosion, and bactericidal behavior are potentially improved, as preferential sites for crack nucleation, attack, and bacterial proliferation are avoided. In addition, much evidence has been reported on the release of Ti particles from Ti implants depending on a wide range of factors such as pH, temperature, dietary and bacterial populations.^79^ In this context, coating with poly(acrylic acid)-based hydrogels could limit or diminish the liberation of metal particles, preventing bone inflammation and the particles reaching other organs.^80^ In this sense, novel poly(acrylic acid)-based biodegradable hydrogels were developed with an adequate balance among degradability, antimicrobial behavior, and osseointegrative capacity that allows them to have good infiltration and adhesion to the implant’s surface.

### 3.1 Synthesis and Characterization of the Cross-Linker

**Figure 5:**
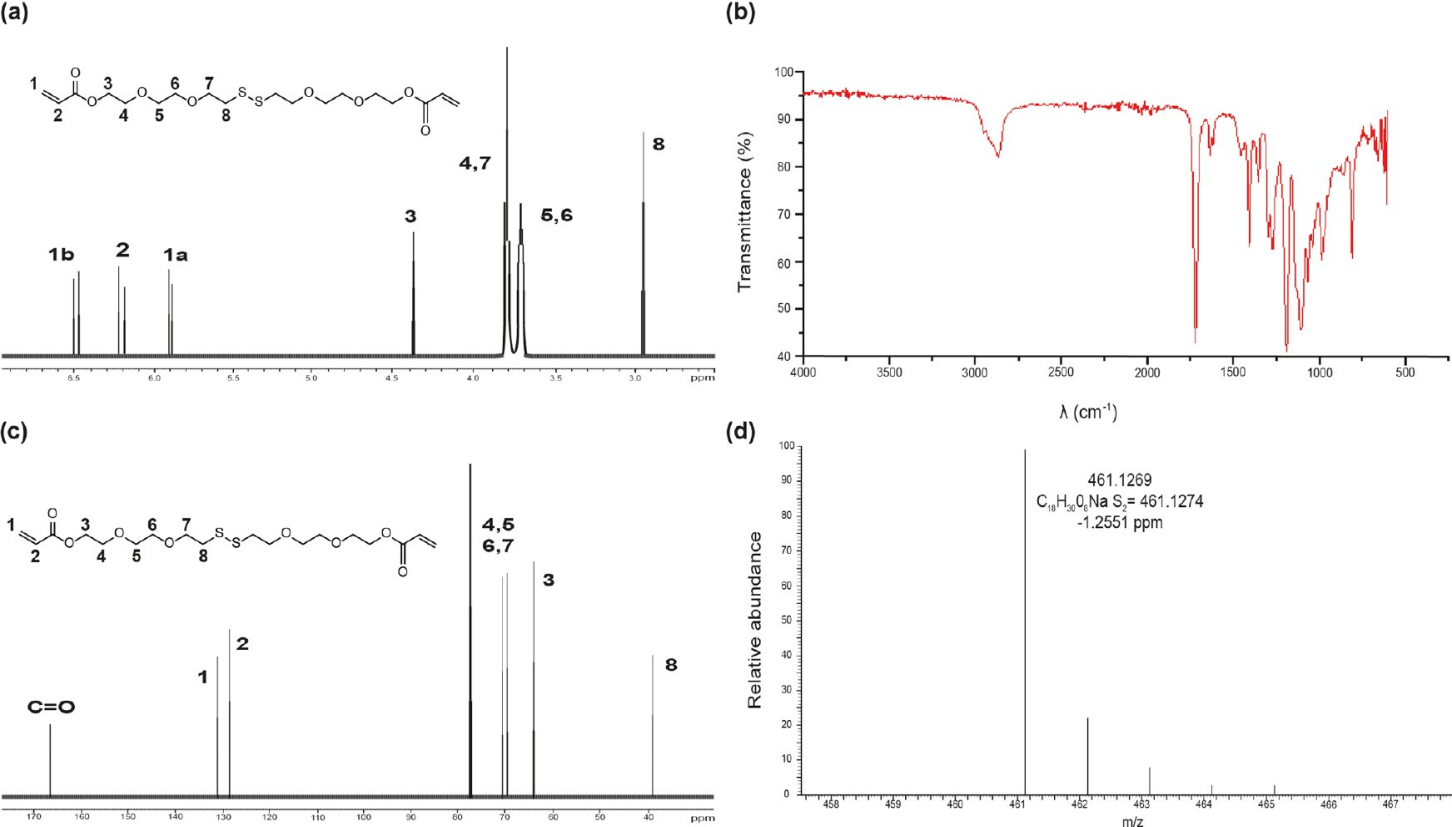
Chemical characterization of 3,6,13,16-tetraoxa-9,10-dithiaoctadecane-1,18-diyl diacrylate (**4**): (a) ^1^H-NMR, (b) FTIR, (c) ^13^C-NMR, and (d) mass spectrum.

To prepare biodegradable poly(acrylic acid)-based hydrogels, a new glutathione-sensitive cross-linker 3,6,13,16-tetraoxa-9,10-dithiaoctadecane-1,18-diyl diacrylate (4) was synthesized containing disulfide bonds. Figure 2 shows the synthetic route followed to obtain **4**. Initially, compounds **2** and **3** were prepared according to the previously described method published by Lang et al.,^70^ with some implementations. Briefly, the reaction between (2-(2-(2-chloroethoxy)ethoxy)ethan-1-ol) (1) and NaHS led to the corresponding thiol **2** as previously described, but distillation as purification methodology was replaced by a chromatographic column using DCM–MeOH (50:1), as eluent, with a quantitative yield (99%, higher than 55% previously described by Lang et al.^70^). Then, an oxidation reaction was performed to generate the diol containing disulfide group **3** with a yield similar to that previously described in the literature, but a chromatography column was not needed to purify the product. Data obtained from the characterization of both compounds were in concordance with those already published. The reaction of **3** with acryloyl chloride led to the novel cross-linker **4** as a yellowish syrup with a yield of 52%. The new monomer was characterized by FTIR, mass spectroscopy, ^1^H NMR, and ^13^C NMR (Figure 5), demonstrating the presence of the disulfide bond and both acrylate groups, as well as the polyoxygenated backbone.

### 3.2 Synthesis and Characterization of Poly(acrylic acid)-Based Polymers (Dry Polymeric Materials)

**Figure 6:**
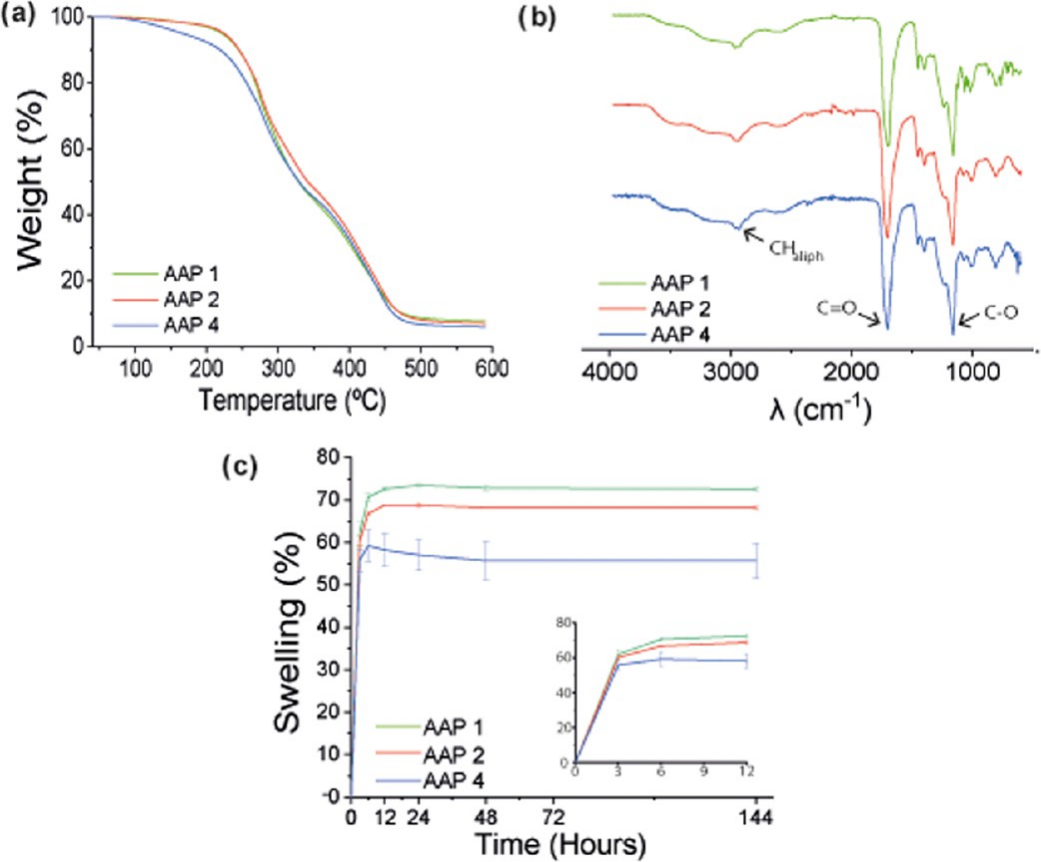
(a) TGA and (b) FTIR of poly(acrylic acid)-based polymers AAP1, AAP2, and AAP4, (c) evolution of hydrogel weight during the swelling test. Gained weight is expressed as a percentage of original dry polymer weight against time expressed in hours.

Since poly(acrylic acid) is not degradable in the human body, it requires a labile moiety to be converted into degradable forms^81,82^ once it forms a hydrogel. For this purpose, the cross-linker **4** was designed to include a disulfide bond that can be broken through a reduction reaction by the biomolecule glutathione. The inclusion of this cross-linker into the polymer backbone would have two objectives: on the one hand, it would add labile points and transform the poly(acrylic acid)-based material into degradable ones by the human body, so that the hydrogel could be degraded as the osseointegration occurs; on the other hand, cross-linking the linear chains of poly(acrylic acid) would allow increasing the potential of the material to act as a hydrogel, absorbing high amounts of water. Both aspects, biodegradability and swelling, could be adjusted by controlling the proportion of the cross-linker. In this sense, the polymers AAP1, AAP2, and AAP4 were obtained under inert atmosphere by radical polymerization of acrylic acid and different proportions of **4** (1, 2, and 4% w/w, respectively), applying AIBN (1% w/w) as the thermoinitiator. AIBN and cross-linker were both dissolved in acrylic acid. All hydrogels were obtained as transparent white-yellowish rubbery materials and characterized (Figure 6).

FTIR showed the presence of acid groups (∼3190 cm^–1^), an aliphatic chain (∼2934 cm^–1^), and carbonyl groups (∼1702 and ∼1158 cm^–1^) in all polymers, demonstrating the formation of polymeric chains. TGA also displayed a similar profile of the three-step decomposition process for all polymers (Figure 6a and Table 1), with a weight loss of approximately 30% for the first step, 25% for the second, and 40% for the last step, proving that the inclusion of different proportions of cross-linkers did not influence the thermal behavior of the polymers. These results are completely in concordance with the thermal behavior shown by commercial poly(acrylic acid). McNeill et al.^83^ demonstrated that poly(acrylic acid) presents two degradation steps centered at approximately 290 and 420 °C that correspond to dehydration of acid groups and decarboxylation, and chain scission. In this sense, the third decomposition step appearing in the cross-linked polymer would correspond to the scission and decomposition of the disulfide cross-linker.

**Table 1:** TGA Data of Polyacrylic Acid-Based Polymers AAP1, AAP2, and AAP4^a^

polymer	^o^*T*_d_ (°C)	^max^*T*_d_ (°C)	Δ*W* (%)
AAP1	244	273/295/412	30/23/37
AAP2	245	276/324/420	24/28/40
AAP4	219	259/307/430	34/21/37

a ^o^*T*_d_: Onset decomposition temperature corresponding to 10% of weight loss; ^max^*T*_d_: maximum rate decomposition temperatures; Δ*W*: weight lost at the corresponding decomposition step.

### 3.3 Swelling Tests of Poly(acrylic acid)-Based Polymers

Since the polymers are infiltrated in the metallic substrates as swelled hydrogels, the swelling capacity of the previously prepared polymeric materials was investigated. Polymeric materials were immersed in water, and their weights were measured at predetermined time intervals. According to eq 1, the swelling capacity of acrylic acid-based polymers was slightly reduced with increasing cross-linking degree due to a reduction in the mobility of the polymeric chains. Nevertheless, the amount of water absorbed by these materials was quite high in all cases, approximately ranging from 60 to 75% of the total dry polymer weight for polymers AAP1–AAP4 (Figure 6c). The swelling profile was similar for all polymers with slight variations. Polymer AAP1 with the highest water absorption capacity (74%) reached its maximum weight in 24 h, while polymer AAP4 with the lowest water absorption capacity (59%) reached its maximum 6 h after the experiment started. Polymer AAP2 showed an intermediate profile between the other two materials, with a maximum absorbing capacity of 69% reaching within 12 h.

### 3.4 Synthesis of Poly(acrylic acid)-Based Hydrogels

Hydrogels AAH1, AAH2, and AAH4 were prepared following the same synthesis procedure previously described but adding an amount of water equal to the volume absorbed for each polymer in the swelling experiments; thus, the following characterization would be conducted on the materials as they were going to be infiltrated to avoid a considerable increment of volume of the hydrogels once inside the substrate pores.

### 3.5 Degradation of Hydrogels in a Reductive Environment

**Figure 7:**
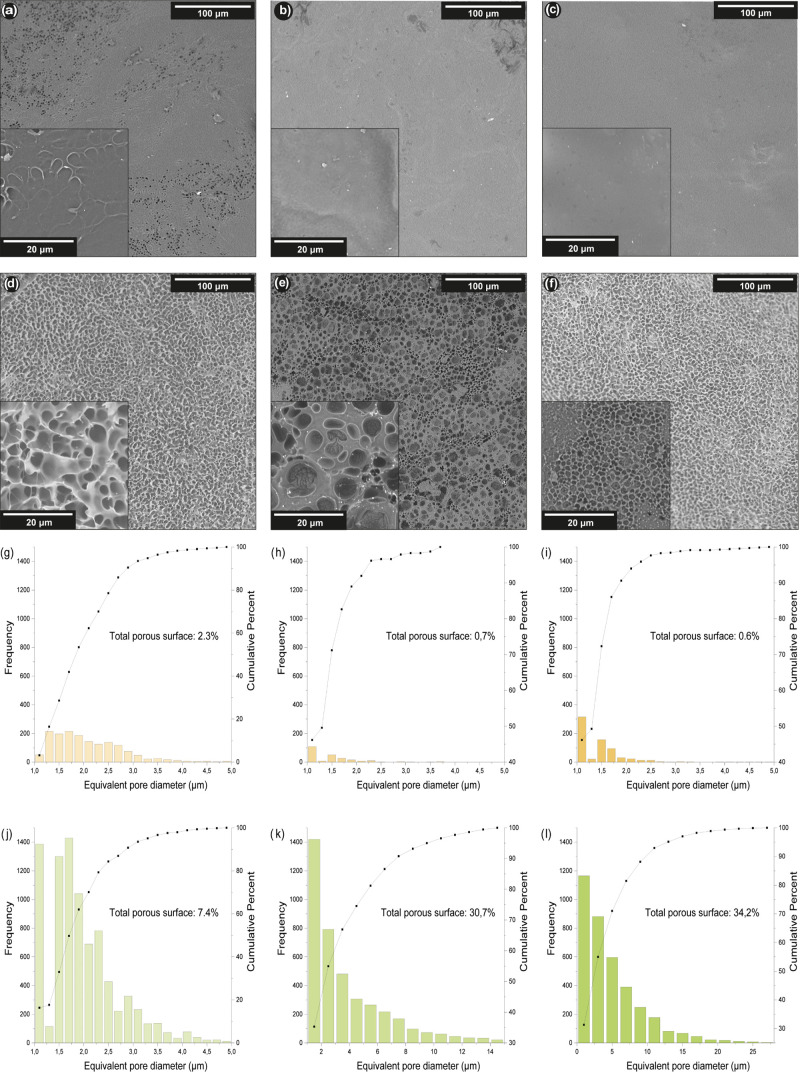
SEM images (a), (b), and (c) show the surfaces of hydrogels AAH1, AAH2, and AAH4 samples, respectively, at the beginning of the test. Micrographs (d), (e), and (f), respectively, show the surfaces of the previously mentioned hydrogels after 28 days of being submerged in 10 mM GSH solution. Histograms (g), (h), and (i) correspond to the pore size distribution of hydrogels AAH1, AAH2, and AAH4 before the degradation study, while histograms (j), (k), and (l) correspond to the same materials after 28 days of degradation in the presence of GSH.

As mentioned above, one of the final objectives of hydrogels is to act as coatings to enhance osteoblast adhesion and proliferation on the implant surface. However, the polymeric chain is required to be eliminated simultaneously with the osseointegration. Therefore, the hydrogels were designed to be degradable, in this case, by the ubiquitous biomolecule glutathione, which acts as a redox buffer in cells, and its reduced form is able to disrupt disulfide bonds such as the group in the newly synthesized cross-linker. Thus, GSH has been used to produce a degradative environment for the hydrogels. On the other hand, the inclusion of the cross-linker would entail the generation of low-molecular-weight polymeric chains that could be excreted by the kidney, with a threshold set at approximately 60 kDa.^84−86^ SEM images were taken at the beginning of the experiment and after 28 days (Figure 7).

On day 0, when samples have not been under the action of GSH yet, micrographs showed a similar plain surface for every hydrogel. However, two facts could be observed after the degradation test: after 28 days of immersion, the surface of each sample turned completely irregular, and a direct relation was observed between the amount and size of pores and the degree of cross-linking of each hydrogel. An eroded surface can be explained by the action of GSH; its reducing power allows it to break the disulfide bond present in the cross-linking agent, separating and releasing poly(acrylic acid) chains, and modifying the surfaces of the hydrogels. As it is the only differential factor between the hydrogels, it can be stated that variations in degradation degrees through the samples in a specified period depend on the proportions of cross-linked agent employed in their polymerization. Thus, a higher proportion of the cross-linking agent (4% w/w) leads to a more altered surface, which means a faster degradation rate, while a lower proportion of the cross-linking agent (1% w/w) leads to a less altered surface, which means a slower degradation rate. We suggest that, as GSH breaks down disulfide bonds at a constant rate, high-cross-linked hydrogel polymeric chains are separated faster (GSH degradation produces lower chains) than low-cross-linked hydrogel ones, inducing bigger and numerous pores in hydrogel AAH4 in comparison to the smaller and less quantitative pores in hydrogel AAH1. The integrity and consistency of some hydrogel AAH4 samples changed significantly at the end of the experiment.

A study of the evolution of porosity in the hydrogels during the degradation process was also conducted. As displayed in Figure 7g–i, initially, the porosity of the hydrogel AAH1 was slightly higher than the ones of the other hydrogels, with a 95% of pores below 3.2–3.4 μm, while the values shown by AAH2 and AAH4 were 2.0–2.2 μm in both cases. In addition, the total porous surface was also higher for AAH1 (2.3%) than for AAH2 (0.7%) and AAH4 (0.6%). However, after 28 days of degradation in the presence of GSH, hydrogel AAH4 presented the biggest pore size, with 95% of pores below 12–14 μm and a total porous surface of 34.2%. The hydrogel AAH2 exhibited 95% of pores below 9–10 μm with a total porous surface of 30.4%. And AAH1 displayed the smallest porosity with 95% of pores below 3.2–3.4 μm with a total porous surface of 7.4%. These results were in concordance with the qualitative estimation performed by using the SEM micrographs.

### 3.6 Wettability Studies of Hydrogels

**Figure 8:**
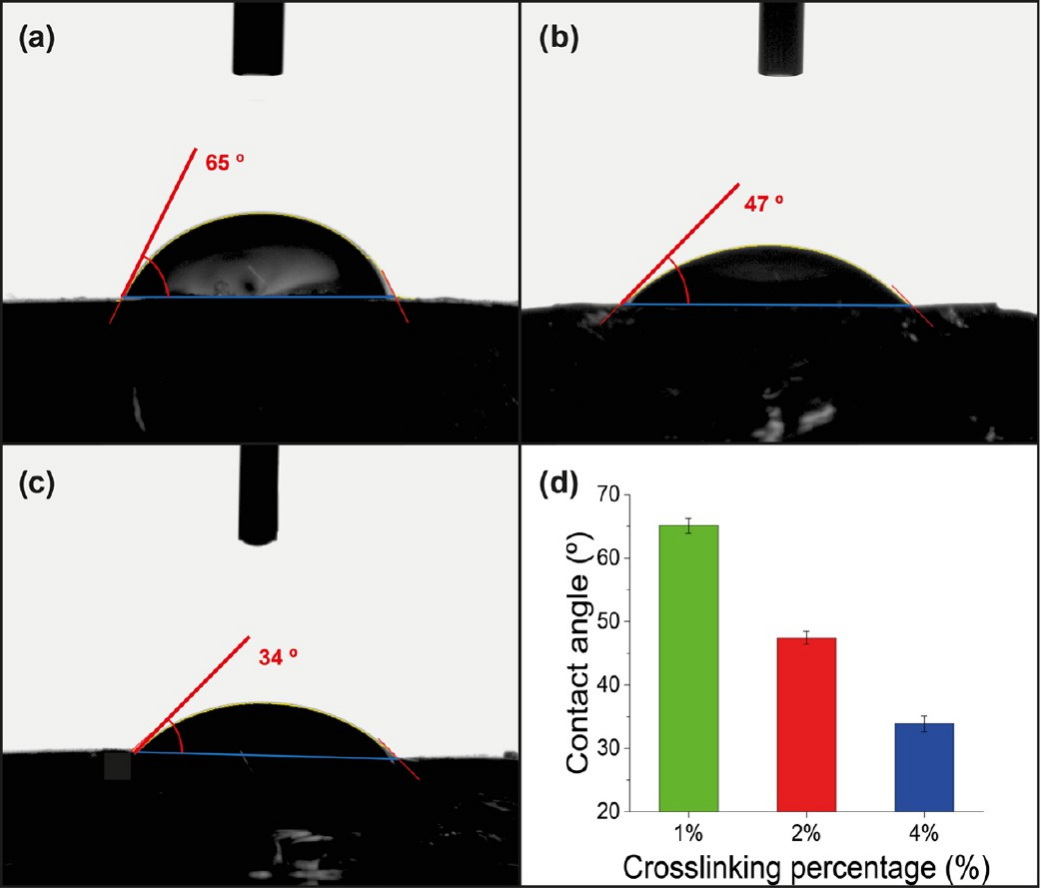
Static contact angle of a waterdrop and surfaces of (a) hydrogel AAH1, (b) hydrogel AAH2, and (c) hydrogel AAH4. (d) Graphical comparison of static contact-angle measurements for each hydrogel.

Wettability (hydrophobicity–hydrophilicity balance) of a material is related to both its potential antibacterial effect and the ability to bind inorganic elements to its surface and promote early-stage mineralization processes.^87,88^ Contact-angle data obtained for each hydrogel were 65.1 ± 1.2° for the hydrogel AAH1, 47.4 ± 1.1° for the hydrogel AAH2, and 33.9 ± 1.2° for the hydrogel AAH4 (Figure 8). According to measurements, a relation between cross-linking degree and a minor static contact angle could be established, where a higher hydrophilicity is associated with a higher cross-link. These results set the foundations for upcoming hydroxyapatite formation studies, where the potential capacity of hydrogel AAH1 to infiltrate and induce osseointegration was tested against hydrogel AAH4 as a representative of lower and higher hydrophilic materials, respectively.

### 3.7 Characterization of Porous c.p. Ti Substrates

Once the porous Ti substrates were fabricated following the process previously described in the experimental section, they were characterized in terms of porosity (total *P*_T_ and interconnected *P*_i_), pore size (*D*_eq_), and morphology (*F*_f_), and macromechanical behavior (Table 2).

**Table 2:** Experimental Porosity Parameters and Estimated Macromechanical Behavior of Porous Implants

	Archimedes’ method		image analysis		macromechanical behavior	
space-holder technique	*P*_T_ (%)	*P*_i_ (%)	*D*_eq_ (μm)	*F*_f_	*E*_d_ (GPa)	σ_*y*_ (MPa)
30 vol %	30.5 ± 0.3	18.4 ± 0.2	190 ± 115	0.73 ± 0.2	55 ± 0.8	350 ± 25
100–200 μm						
60 vol %	58.3 ± 0.6	53.40 ± 0.9	393 ± 130	0.78 ± 0.4	31.2 ± 1.5	87 ± 32
355–500 μm						

According to the results displayed in Table 2, the total porosity of both substrates was very similar to the intended one, pointing out the space-holder technique as an excellent candidate to fabricate porous substrates. As expected, the interconnected porosity was much higher for the substrate with 60 vol % porosity (53.4%) than for the one with 30 vol % (18.4%). These values would indicate that the substrates with 60 vol % porosity would favor more effectively the bone in-growth and vascularization processes. In addition, although in both cases Young’s modulus was reduced, stiffness of substrates with 60 vol % porosity was closer to the natural bone one (31.2 GPa, against 55 GPa shown by the substrates with 30 vol %). However, the mechanical resistance of the substrate with 30 vol % porosity presented much better values (350 MPa) than the other substrates (87 MPa).

In summary, for the substrate with 30 vol % porosity and 100–200 μm pore size distribution, although in terms of yield strength it is the best since it guarantees the requirements of the cortical bone tissue (150–180 MPa), the decrease in *E*_d_ is still insufficient to completely solve the stress-shielding phenomenon. On the other hand, the content, degree of interconnection and size of the pores, is less attractive to promote bone in-growth and favor biopolymer infiltration. On the other hand, the substrate with 60 vol % porosity and 355–500 μm pore size distribution favors vascularization and infiltration, as well as generates *E*_d_ values closer to that of cortical bone (20–25 GPa). But, however, its mechanical resistance is compromised.

### 3.8 Evaluation of the Antibiofouling Capacity of Poly(acrylic acid)-Based Hydrogels

As previously mentioned, to determine the potential antibacterial behavior of the hydrogels, an optimized methodology based on the capacity of the materials to avoid bacterial attachment to their surfaces was developed. Coating materials whose antibacterial capacity is inherent to their composition represent a new step in novel techniques to avoid the use of antibiotics to reduce the appearance of microbial-related infections, but there is a lack of information about standardized methodologies employed to test their efficiency due to their heterogeneity. Due to this inconvenience, a methodology based on an existing bibliography was tuned and set up, taking into account the malleability of our materials.^57^ Its application showed that the number of CFU of *P. aeruginosa* and *S. aureus* developed on the surface of every hydrogel was below the lower limit that this technique is able to sense (<3 × 10^2^ CFU/cm^2^), and no colonies developed on the surface of agar plates in every single test. These results indicated that hydrogels completely inhibited the bacterial growth of these species. On the other hand, blank samples (c.p. Ti substrates) showed bacterial growth on their surface, (4.6 ± 0.7) × 10^5^ CFU/cm^2^ for *P. aeruginosa* and (3.4 ± 0.8) × 10^5^ CFU/cm^2^ for *S. aureus*.

### 3.9 Visualization of Bacterial Attachment to Surfaces

**Figure 9:**
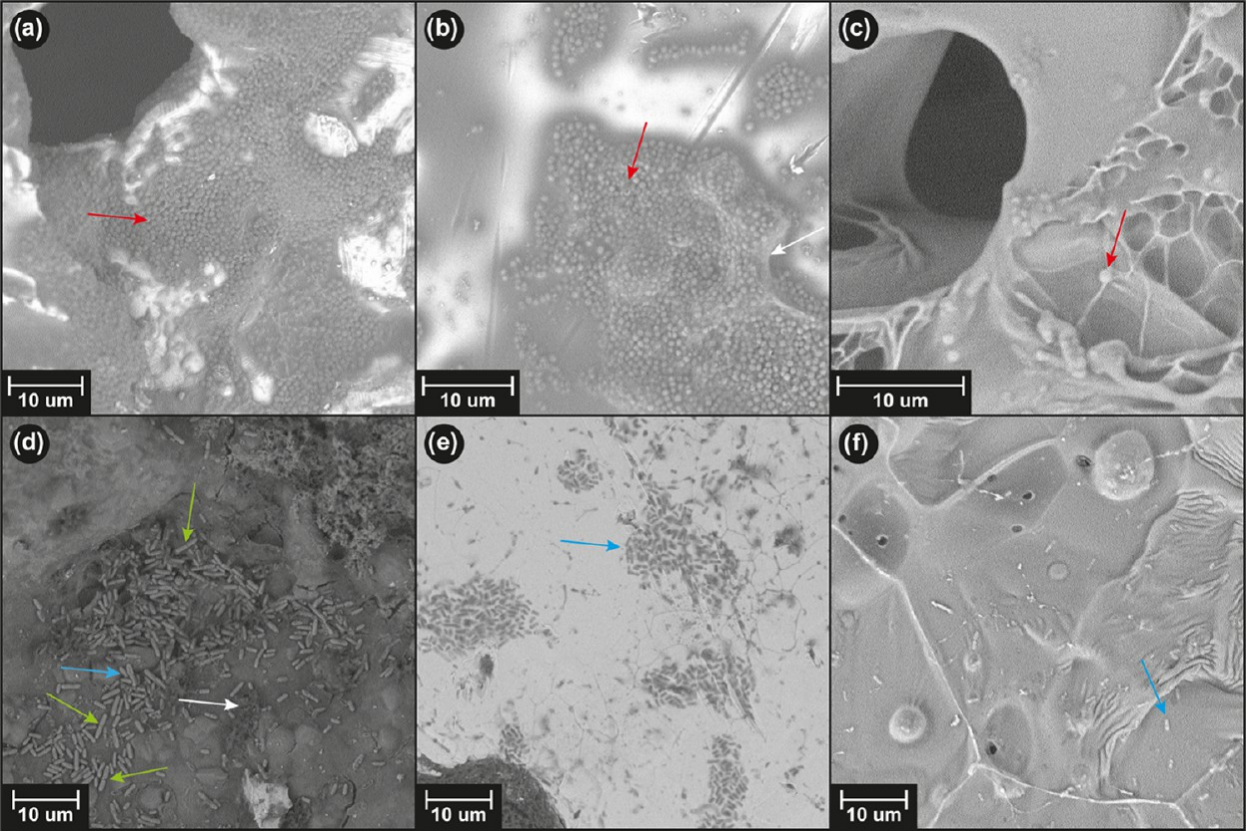
Low-vacuum SEM pictures of c.p. Ti substrates with 30% porosity and 100–200 μm pore size distribution, c.p. Ti substrates with 60% porosity and 355–500 μm pore size distribution and hydrogel AAH4, respectively, cultured with (a–c) *S. aureus* and (d–f) *P. aeruginosa*. Red arrows mark *S. aureus* clumps, blue arrows point to the accumulation of *P. aeruginosa*, white arrows indicate the organic material secreted by bacteria, and green arrows signal dividing bacteria.

Figure 9 shows low-vacuum SEM micrographs of c.p. Ti substrates with 30% porosity and 100–200 μm pore size distribution (Figure 9a), and c.p. Ti substrates with 60% porosity and 355–500 μm pore size distribution (Figure 9b), respectively, were cultured with *S. aureus*. The accumulation of bacteria was visible all over the substrate surface (red arrows). It is possible to observe large colonies of bacteria with spherical morphology corresponding to the *cocci* of *S. aureus*, which formed a biofilm onto the surface of both Ti surfaces. Similarly, Figure 9d,e displays *P. aeruginosa* covering the surface of the same substrates previously mentioned (blue arrows). In this case, the morphology observed corresponded to long rods, typical of *P. aeruginosa*. As before, the bacteria were colonizing the surface and producing a biofilm onto it. The observation of some bacteria in pairs (pointed with green arrows) corresponded to bacteria that were dividing at that precise moment, indicating that active multiplication was taking place. In addition, white arrows pointed to the organic extracellular material, highlighting their high adhesion and proliferation. However, Figure 9c,f presents, as an example, micrographs of the hydrogel AAH4 cultured in the presence of the same bacterial strains, *S. aureus* and *P. aeruginosa*, respectively. In these cases, although some bacteria were visible, they were isolated and in an extremely reduced number. These images demonstrated that the bacterial growth on top of the untreated c.p. Ti substrates was much higher (three orders of magnitude) than over the hydrogel surface, which showed bacterial attachment below the detection threshold of the technique.

A gap of three orders of magnitude in the results demonstrates with no doubt that hydrogel coatings would perform better at preventing bacterial colonization rather than nude c.p. Ti substrates. Different explanations can explain these results. On one hand, the surface of the uncoated Ti material is very porous, and the topology can create deep hollows where bacteria can be entrapped and biofilms developed.^89^ However, the surface of the material after coating with acrylic polymers with smaller pores can prevent or diminish bacterial entrapment and biofilm formation. On the other hand, the composition of the acrylic polymer is quite inert for bacteria and only some environmental strains are capable of partial biodegradation.^90^

### 3.10 Infiltration of Porous Ti Substrates

**Figure 10:**
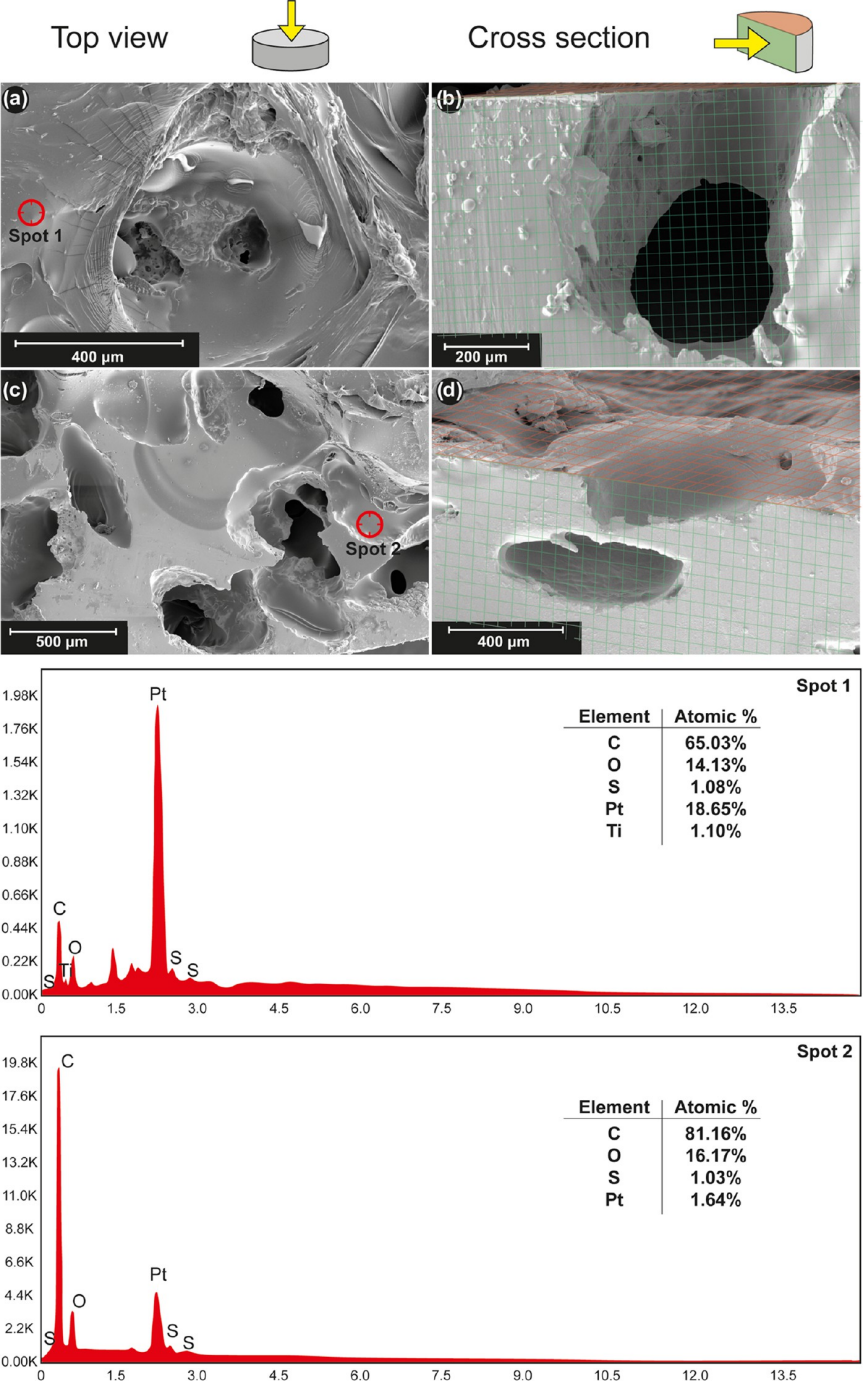
SEM images from infiltrated hydrogels AAH1: (a) top view and (b) cross-sectional view of 30 vol % porosity and 100–200 μm average pore size c.p. Ti samples and AAH4: (c) top view and (d) cross-sectional view of 60 vol % porosity and 355–500 μm average pore size c.p. Ti substrates. EDS images show the presence of the hydrogels through the high amount of atomic % of C in two different points of the substrate surface.

The authors of this article have extensive experience in the fabrication of porous c.p. Ti substrates.^5,8,57,63,66,77^ They have already demonstrated that to obtain a good biomechanical balance, the substrate porosity must range between 30 and 60 vol %, while the pore size distribution must oscillate between 100 and 200 μm and 355–500 μm. Obviously, the use of specific characteristics will depend on the global requirements to be achieved. In this sense, a lower porosity and pore size distribution will increase mechanical resistance, while higher porosity and pore size distributions will improve the functional behavior of the implant and the infiltration and adhesion of coatings. Therefore, in this research work, two extreme characteristics have been selected and investigated for the coating of hydrogels onto Ti substrates: 30 vol % porosity and 100–200 μm average pore size, and 60 vol % porosity and 355–500 μm average pore size. In addition, the infiltration process was also tested by applying hydrogels AAH1 and AAH4. Since antibacterial tests demonstrated that all hydrogels had growth inhibition activity, hydrogels AAH1 and AAH4 were selected as those with lower and higher hydrophilia. Infiltration of the hydrogel on top of the substrate surface was performed via direct synthesis. Albeit every single polymerization was successful, 30 min of heating (instead of 15 min previously described) was needed to completely polymerize. A heat-shrinking tube was employed to prevent the mixture from penetrating the substrates through side pores and control the quantity of polymer needed. After polymerization, the SEM images showed the polymer coating, which was corroborated by elemental composition analysis (Figure 10). Polymer coating within the pores was also observed in every sample, which means that infiltration was correctly performed.

### 3.11 Hydroxyapatite Formation on Coated Substrates

**Figure 11:**
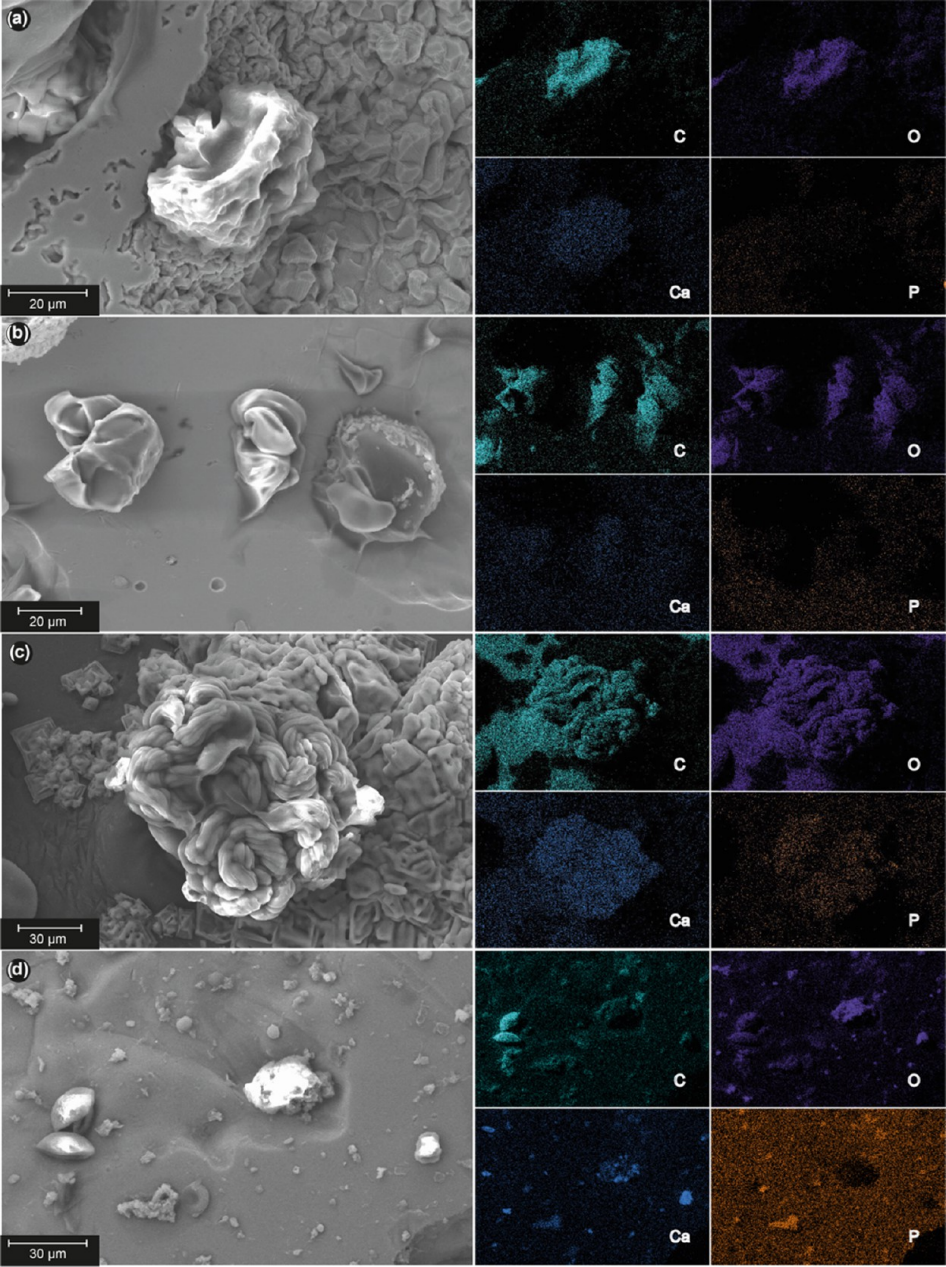
SEM images and element mapping after osseointegration experiments of the surfaces of (a, b) hydrogel AAH1 and (c, d) hydrogel AAH4 infiltrated on c.p. Ti substrates with 30 vol % porosity and 100–200 μm average pore size (a, c), and 60 vol % porosity and 355–500 μm average pore size (b, d), respectively.

Previous studies state that implants with proper osseointegration lead to a higher biocompatibility and lower rejection and loosening. For this reason, it is usual to find osseointegration inductors as coating materials, such as hydroxyapatite nanoparticles^91,92^ or bioactive glasses.^8^ However, the effect of pH on the formation of hydroxyapatite precursors has already been demonstrated.^8^ Therefore, since the previously prepared hydrogels are based on polyanionic polymers, the potential formation of hydroxyapatite induced by the hydrogels themselves was investigated. In this sense, the potential hydroxyapatite-forming ability of these hydrogels was tested to determine whether they produce this induction or not and which material would be preferred as a coating for c.p. Ti substrates. Infiltration of hydrogels AAH1 and AAH4 in c.p. Ti samples with 60 vol % porosity and 355–500 μm average pore size and 30 vol % porosity and 100–200 μm average pore size were once again confirmed in this experiment. Figure 11 shows a composition of these SEM and elemental analyses of C, O, Ca, and P images. Elemental composition analyses of the images showed structures of a polymeric nature (C and O), which could be observed all over the surfaces of every sample. Furthermore, Cl, Na, Ca, and P species, which were provided by the SBF solution, were deposited onto the surface. The deposition of Ca and P atoms has been associated with higher osseointegration as it indicates the nucleation that precedes the hydroxyapatite formation process.^93^

In addition, these depositions seem to be preferably located onto polymeric structures, which indicates the ability of polymeric compounds to initiate a nucleation process for Ca and P and promote the appearance of hydroxyapatite species, as can be seen by analysis of the elemental composition in all of the pictures in Figure 10. This fact enhances the desired coating performance as it suggests that polymers induce the formation of hydroxyapatite species and, consequently, a better osseointegration process. However, some differences have been observed between hydrogel AAH1 and hydrogel AAH4 hydroxyapatite formation capacities; the nucleation process is more favored in higher cross-linked samples rather than lower cross-linked ones, as can be observed in images (c) and (d), which can be related to the lower static contact-angle data previously obtained. It can be concluded that hydrogel AAH4 has the highest osseointegration capacity among candidates.

## 4 Conclusions

This research work presents a double-synergic approach to potentially enhance the performance of metallic implants. On the one hand, the use of porous c.p. Ti was suggested for better biomechanical behavior. Porous substrates were fabricated via the space-holder technique, modifying not only the amount of porosity (30 and 60 vol %) but also the particle size distribution (100–200 and 355–500 μm) of the holder. In both cases, Young’s modulus was reduced, although the reduction was more significant for the substrates with 60 vol % porosity and 355–500 μm pore size distribution. However, substrates with 30 vol % pore content and 100–200 μm average pore size presented the best mechanical resistance. On the other hand, the application of novel chemically degradable polymeric coatings was proposed to improve osseointegration, avoiding bacterial-related infections. A new diacrylate cross-linker was employed to prepare acrylic acid–based polymers in different proportions (1, 2, and 4%), leading to the corresponding cross-linked polymers (AAP1, AAP2, and AAP4). The swelling behavior of polymeric materials allowed turning them into poly(acrylic acid)-based hydrogels (AAH1, AAH2, and AAH4). Wettability tests demonstrated a general trend of hydrophilicity for the three materials, although a slight increment was observed when increasing the cross-linking degree. The three hydrogels showed outstanding properties as antibacterial materials when tested against strains of Gram-(−) *P. aeruginosa* and Gram-(+) *S. aureus*. Furthermore, they prevented the formation of bacterial biofilms onto coated surfaces, which is a dangerous situation, particularly in hospital (nosocomial) infections. Hydrogels AAH1 and AAH4 with lower and higher hydrophilicity, respectively, were infiltrated in the porous c.p. Ti substrates to investigate their infiltration and adhesion capacity, showing good surface coating, adhesion, and infiltration inside the inner pores. Finally, they were tested to explore their potential osseointegration properties. Although the formation of hydroxyapatite was observed in all samples, the hydrogel AAH4 infiltrated in porous c.p. Ti with 30 vol % porosity and 100–200 μm pore size distribution exhibited the best osseointegrative capacity. Therefore, the combination of these results provided a means of combining the best mechanical resistance of Ti substrates with 30 vol % porosity bearing 100–200 μm pore size distribution with the antibacterial infiltrated hydrogel AAH4 as the best tandem to be applied for alternative treatments that require the use of implants in bone injuries.
